# Changes in Structural Neural Networks in the Recovery Process of Motor Paralysis after Stroke

**DOI:** 10.3390/brainsci14030197

**Published:** 2024-02-21

**Authors:** Ikuo Kimura, Atsushi Senoo, Masahiro Abo

**Affiliations:** 1 Department of Rehabilitation Medicine, Izumi Memorial Hospital, 1-3-7 Motoki, Adachi-ku, Tokyo 123-0853, Japan; 2 Department of Rehabilitation Medicine, The Jikei University School of Medicine, 3-25-8 Nishi-Shimbashi, Minato-ku, Tokyo 105-8461, Japan; abo@jikei.ac.jp; 3 Department of Radiological Sciences, Graduate School of Human Health Sciences, Tokyo Metropolitan University, 7-2-10 Higashi-ogu, Arakawa-ku, Tokyo 116-8551, Japan; senoo@tmu.ac.jp

**Keywords:** stroke, neurorehabilitation, repetitive transcranial magnetic stimulation, rTMS, diffusion tensor imaging, DTI, structural neural networks, graph theory

## Abstract

In recent years, neurorehabilitation has been actively used to treat motor paralysis after stroke. However, the impacts of rehabilitation on neural networks in the brain remain largely unknown. Therefore, we investigated changes in structural neural networks after rehabilitation therapy in patients who received a combination of low-frequency repetitive transcranial magnetic stimulation (LF-rTMS) and intensive occupational therapy (intensive-OT) as neurorehabilitation. Fugl-Meyer assessment (FMA) for upper extremity (FMA-UE) and Action Research Arm Test (ARAT), both of which reflected upper limb motor function, were conducted before and after rehabilitation therapy. At the same time, diffusion tensor imaging (DTI) and three-dimensional T1-weighted imaging (3D T1WI) were performed. After analyzing the structural connectome based on DTI data, measures related to connectivity in neural networks were calculated using graph theory. Rehabilitation therapy prompted a significant increase in connectivity with the isthmus of the cingulate gyrus in the ipsilesional hemisphere (*p* < 0.05) in patients with left-sided paralysis, as well as a significant decrease in connectivity with the ipsilesional postcentral gyrus (*p* < 0.05). These results indicate that LF-rTMS combined with intensive-OT may facilitate motor function recovery by enhancing the functional roles of networks in motor-related areas of the ipsilesional cerebral hemisphere.

## 1. Introduction

In recent years, researchers have shown that neurorehabilitation can reduce motor paralysis after stroke [1]. A wide variety of approaches are used to facilitate neurorehabilitation including the following: constraint-induced movement therapy (CIMT) not using special equipment [2,3], interventions combining electrical stimulators or magnetic stimulators with conventional therapy [4,5,6], and interventions using large robot-assisted equipment [7,8]. Our study group was the first in the world to develop a combined intervention consisting of low-frequency repetitive transcranial magnetic stimulation (LF-rTMS) and intensive occupational therapy (intensive-OT), and used this combination therapy in a large number of patients with upper extremity motor paralysis after stroke [9]. rTMS is a noninvasive method of stimulating the cerebral cortex, and LF-rTMS is known to locally reduce cortical excitability [10]. LF-rTMS to the contralesional hemisphere is able to enhance indirectly the activity in the ipsilesional hemisphere through decreasing interhemispheric inhibition (IHI) in the contralesional hemisphere [11]. Our data show that LF-rTMS and intensive-OT in combination significantly improves upper limb motor function [9,12,13,14,15]. Although the mechanism underlying this improvement remains largely unknown [1], it is thought that plasticity in the central nervous system is involved [16].

Magnetic resonance imaging including functional magnetic resonance imaging (fMRI) and diffusion tensor imaging (DTI) are used to elucidate functional and structural features of the brain. In recent years, DTI data, in particular, have been correlated with the improvement of post-stroke paralysis [16,17,18,19,20,21,22,23,24]. Applying a mathematical approach, namely, graph theory, to analysis of DTI data allows greater understanding of the structural neural networks of the brain [25,26]. This method is excellent for analyzing not only neural networks within a designated region of interest (ROI), but also neural networks throughout the brain automatically.

Although changes in structural neural networks during recovery from motor paralysis after stroke have been reported [27,28], to the best of our knowledge, only Wanni Arachchige et al. [24] have investigated the impact of LF-rTMS combined with intensive-OT on the structural neural networks in detail. Their study used the same LF-rTMS plus intensive-OT protocol as ours [24]. However, they did not distinguish between left and right hemispheres to increase the number of cases available for analysis, and calculated network measures for only 20 motor-function-related regions in the cerebrum. The current study remedied these shortcomings by dividing patients into right-sided paralysis and left-sided paralysis groups, and calculating network measures for all areas of the brain divided into 84 regions. Our purpose was to investigate changes in whole-brain structural neural networks between patients with right-sided paralysis and patients with left-sided paralysis who received LF-rTMS and intensive-OT in combination.

## 2. Materials and Methods

The current study assessed how rehabilitation therapy changed structural neural networks in the brains of patients who received LF-rTMS combined with intensive-OT in a maintenance rehabilitation facility. Ikuo Kimura was responsible for imaging analysis and outcome assessment.

This study was approved by the Ethics Committee of Izumi Memorial Hospital (protocol code: 29A-1). All patients received explanations about the use of laboratory data obtained during the study period for academic purposes as well as the conduct of LF-rTMS and intensive-OT, and they provided written consent to participate.

### 2.1. Patients

Patients consisted of those who had disease onset 6 months earlier and underwent LF-rTMS combined with intensive-OT in Izumi Memorial Hospital from April 2017 to October 2022. The patients also met all of the following criteria: (1) aged between 18 and 90 years, (2) right-handed, (3) first-ever stroke caused by infarction or hemorrhage, (4) nonbilateral stroke, (5) affected upper limb Brunnstrom recovery stage 3 to 5 [29], and (6) no past history of convulsions. In addition, patients receiving LF-rTMS were excluded if they had contraindications listed in the guidelines proposed by Wassermann as a precondition [30].

### 2.2. Rehabilitation Therapy

The combination therapy was performed as an inpatient treatment in Izumi Memorial Hospital on 15 consecutive days excluding Sundays and consisted of a daily routine of LF-rTMS for 40 min once, face-to-face intensive-OT between a patient and an occupational therapist for 60 min twice, and patient self-training for 60 min twice.

### 2.3. LF-rTMS: Equipment and Protocol

The stimuli were administered using a MagPro R30 stimulator (MagVenture Company, Farum, Denmark) equipped with a 70 mm figure-8 coil according to the following protocol: stimulation site, the primary motor cortex of the contralesional hemisphere; stimulus intensity, 90% of the minimum output that allowed recording of motor evoked potentials (MEPs) from the first dorsal interosseous muscle; stimulus frequency, 1 Hz; number of stimuli, 2400 times/day (40 min/day).

### 2.4. Clinical Assessment of Motor Function

Fugl-Meyer assessment (FMA) for upper extremity (FMA-UE) [31,32] and Action Research Arm Test (ARAT) [33], both of which reflected upper limb motor function, were conducted by an occupational therapist and used as measures of improvement by rehabilitation therapy. Functional assessments were conducted at hospital admission and approximately 2 months later.

### 2.5. MRI Acquisition

Before and after rehabilitation therapy, DTI and three-dimensional T1-weighted imaging (3D T1WI) data were acquired using the 3-tesla MRI scanner (Discovery MR750w 3.0T, GE MEDICAL SYSTEMS) installed in Izumi Memorial Hospital. The timing of MRI data acquisition and upper limb motor function assessment was almost the same. The DTI acquisition conditions were as follows: sequence, spin-echo echo-planar imaging (SE-EPI); b-value, 1000 s/mm^2^; b-vector, 30 directions; repetition time (TR), 15,000 ms; echo time (TE), 96.2 ms; slice thickness, 3 mm; matrix, 256 × 256 pixels; voxel size, 1 mm × 1 mm × 3 mm. Those for 3D T1WI acquisition were as follows: sequence, fast spoiled gradient echo (FSPGR); repetition time (TR), 6.6 ms; echo time (TE), 2.6 ms; slice thickness, 1 mm; matrix, 256 × 256 pixels; voxel size, 1 mm × 1 mm × 1 mm.

### 2.6. Assessment of 3D T1WI

Using 3D T1WI data, we divided patients into left-sided paralysis and right-sided paralysis groups and excluded patients with lesions in the cerebral cortex or brainstem whose data were incomplete. Subsequently, the number of patients included for analysis were 9 with left-sided paralysis and 5 with right-sided paralysis. Clinical characteristics of the patients included for analysis are shown in Table 1.

### 2.7. Development of Structural Neural Networks and Graph Theoretical Analysis

The construction of structural neural networks was based on the analysis of DTI and 3D T1WI data. Distortion of DTI images was corrected (preprocessing) using FSL (https://fsl.fmrib.ox.ac.uk/fsl/fslwiki/, accessed on 22 January 2024). For 3D T1WI images, segmentation and parcellation of brain parenchyma into the respective regions corresponding to those in the Desikan–Killiany Atlas [34] were performed using FreeSurfer (https://surfer.nmr.mgh.harvard.edu/, accessed on 22 January 2024). The Desikan–Killiany Atlas divides the brain structures into 84 regions. Next, the DTI image was brought into register with the 3D T1WI image. Finally, 1 × 10^6^ streamlines were extracted probabilistically (probabilistic tractography) based on DTI data using MRtrix3 (https://www.mrtrix.org/, accessed on 22 January 2024), and an 84 × 84 square structural connectivity matrix representing the connectivity strength between two arbitrary brain regions was generated.

Next, following these numeric evaluations, network measures [25,26] based on graph theory were calculated using GRETNA (https://www.nitrc.org/projects/gretna/, accessed on 22 January 2024) [35]. Four graph-theory-derived network measures were selected: degree centrality, betweenness centrality, nodal clustering coefficient, and nodal efficiency.

### 2.8. Meaning of the Network Measures [25,26,36,37,38]

Graph theory is a mathematical tool for visualizing networks as a collection of nodes and edges. The brain can be divided into 84 regions, each corresponding to a node, and neural connections between these regions, each corresponding to an edge.

**Figure 1:**
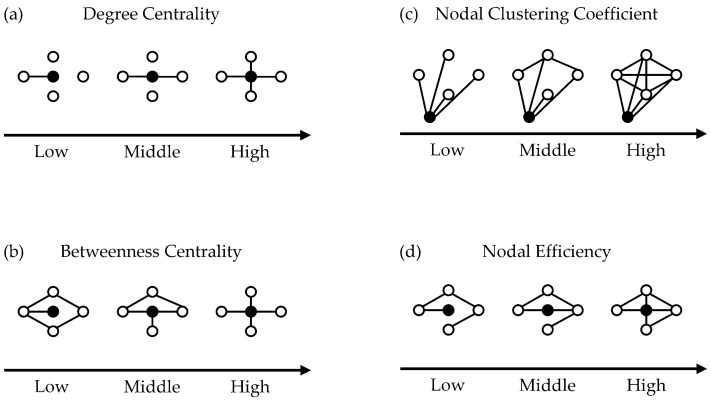
Schematic representation of network measures. (**a**) Degree Centrality; (**b**) Betweenness Centrality; (**c**) Nodal Clustering Coefficient; (**d**) Nodal Efficiency. Circles represent nodes, lines correspond to edges. Black circles represent nodes of interest. This figure was partially excerpted from a figure appearing in [36] Onoda, K. Basis of graph theory on brain imaging study.

Figure 1 shows the network measures schematically.

- Degree centrality is defined as the number of connections to the node of interest.
- Betweenness centrality is defined as the proportion of the nodes of interest existing on the connecting path between nodes other than those of interest. The larger the value is, the more the node functions as a hub in the network.
- Nodal clustering coefficient is defined as the proportion of connections between nodes that are connected to the node of the interest. The larger the value is, the more functionally separate the network is.
- Nodal efficiency is defined as the inverse of the average length of the shortest paths between the nodes of interest and every other node. The larger the value is, the more functionally integrated the network is.

### 2.9. Statistical Analysis

All data were statistically analyzed by R software version 4.0.3 (https://www.R-project.org/, accessed on 22 January 2024). Rehabilitative changes in upper extremity measures (FMA and ARAT scores) as well as network measures were compared between groups using the Wilcoxon signed-rank test. *p* < 0.05 was considered to be statistically significant.

## 3. Results

### 3.1. Changes in Upper Limb Motor Function

In patients with left-sided paralysis, the FMA-UE and ARAT scores (measures of limb motor function) were significantly increased, respectively, from 44.8 ± 14.1 to 49.2 ± 11.2 (*p* = 0.0141) and 26.6 ± 17.1 to 29.6 ± 18.0 (*p* = 0.0220) after rehabilitation therapy. On the other hand, the FMA-UE and ARAT scores were non-significantly increased from 24.0 ± 17.4 to 28.2 ± 17.9 (*p* = 0.0579) and from 9.0 ± 11.8 to 11.0 ± 12.7 (*p* = 0.3710), respectively, in patients with right-sided paralysis and from 37.4 ± 17.9 to 41.7 ± 16.8 (*p* = 0.0016) and 20.3 ± 17.3 to 22.9 ± 18.3 (*p* = 0.0088), respectively, in the entire patient group. The results so far are shown in Table 2.

### 3.2. Generation of Streamlines and the Structural Connectivity Matrix

**Figure 2:**
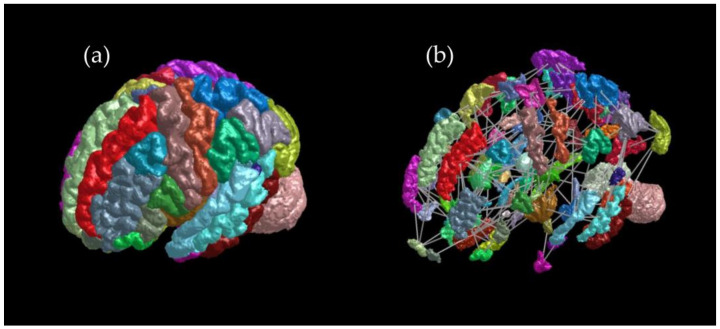
The results of (**a**) parcellation, division of the brain structure into 84 regions and (**b**) node and edge visualization. Different regions are shown with different colors, and the colors themselves have no meaning.

**Figure 3:**
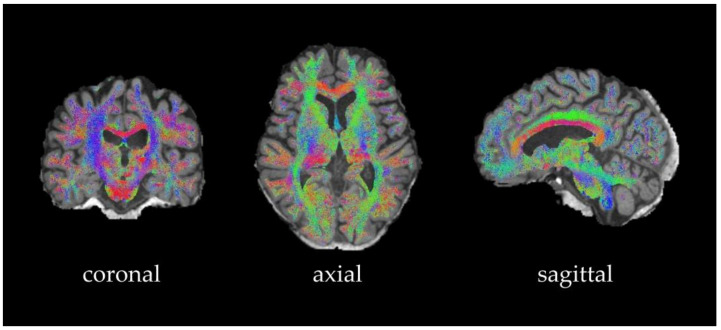
Results of streamline creation, displayed as an overlay on the 3D T1W image. The tracks are color-coded. Red indicates streamlines from right to left, green from anterior to posterior, blue from superior to inferior.

**Figure 4:**
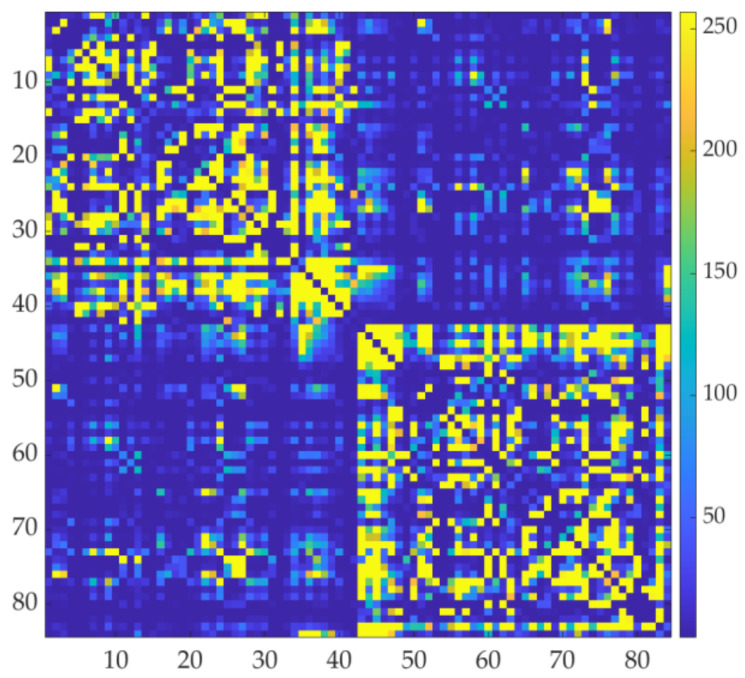
Structural connectivity matrix. The vertical axis and horizontal axis correspond to 84 regions in the divided brain structure. Different colors are used to represent the connectivity strength of pathways between regions.

The brain parenchyma, which had been created based on 3D T1WI data using FreeSurfer, was divided into 84 regions corresponding to those in the Desikan–Killiany Atlas (Figure 2a). Streamlines were extracted probabilistically from a DTI data set using MRtrix3 (example shown in Figure 3). Structural connectivity matrices, with connectivity strength between brain regions indicated by different colors, were generated. The data used to prepare Figure 2, Figure 3 and Figure 4 are from one patient in the LF-rTMS group (an 81-year-old female, after left thalamic hemorrhage).

### 3.3. Changes in Network Measures

Table 3 shows brain regions where individual network measures were statistically significantly changed by rehabilitation treatment (*p* < 0.05). An increase is indicated by green letters, while a decrease is indicated by blue letters.

In patients with right-sided paralysis, no significant changes in network measures occurred.

On the other hand, in patients with left-sided paralysis, the degree centrality and nodal efficiency were significantly increased in the ipsilesional banks of superior temporal sulcus (banks of STS), isthmus of the cingulate gyrus, and paracentral lobule, while they were significantly decreased in the ipsilesional postcentral gyrus. The betweenness centrality was significantly increased in the ipsilesional banks of STS and isthmus of the cingulate gyrus with the significant decrease in the postcentral gyrus, while it was significantly increased in the contralesional caudal anterior cingulate cortex. The nodal clustering coefficient was significantly decreased in the ipsilesional isthmus of the cingulate gyrus with a significant increase in the postcentral gyrus, while it was significantly increased in the contralesional caudal anterior cingulate cortex. All network measures had changed significantly in the ipsilesional isthmus of the cingulate gyrus and postcentral gyrus.

## 4. Discussion

To the best of our knowledge, no study has assessed and shown changes in whole-brain structural neural networks between patients with right-sided paralysis and patients with left-sided paralysis who received LF-rTMS and intensive-OT in combination in a maintenance rehabilitation facility [22,24]. Among the studies evaluating structural and functional changes in the brain based on DTI data, some studies did not distinguish between left and right hemispheres, but rather between ipsilesional and contralesional hemispheres to increase the number of cases available for analysis [20,21,22,24]. The only data available to us for analysis were from patients with left-sided paralysis. Thus, it was impossible to compare changes in the structural neural networks between patients with right-sided paralysis and patients with left-sided paralysis, as we had initially planned.

In contrast to our study (Table 3), a prior investigation of the impact of LF-rTMS plus intensive-OT on structural neural networks by Wanni Arachchige et al. [24] reported a completely different conclusion. Inconsistencies between our results and the prior study’s results may be because Wanni Arachchige et al. calculated network measures for only 20 motor-function-related regions in the cerebrum (caudate, cerebellum, cuneus, frontal pole, pallidum, postcentral gyrus, precentral gyrus, putamen, superior parietal, thalamus in the ipsilesional and contralesional hemisphere) as previously described [27,39,40,41]. Thus, their study did not include regions such as the isthmus of the cingulate gyrus, banks of STS, caudal anterior cingulate cortex, and others, in which significant changes occurred in our study. Therefore, the validity of a simple comparison between these study findings and our findings is questionable.

Regarding changes in network measures in patients with left-sided paralysis, our study showed that the ipsilesional isthmus of the cingulate gyrus tended to have an increased connectivity with other regions, as well as act as a network hub strengthening functional connectivity and functional integration in the entire network. On the other hand, the ipsilesional postcentral gyrus tended to have decreased connectivity with other regions, as well as act as a hub that weakens functional connectivity and creates functional separation. In the regions with a significant change in some network measures, the ipsilesional banks of STS tended to have an increased connectivity with other areas and act as a network hub that strengthens functional connectivity, while the contralesional caudal anterior cingulate cortex tended to act as a hub that weakens functional connectivity and creates functional separation. Obviously, changes in structural neural network connectivity differed between the prior study by Wanni Arachchige et al. and our study. The prior study [24] showed increased connectivity in individual regions both in ipsilesional and contralesional hemispheres. On the other hand, our study showed increased connectivity in regions predominantly in the ipsilesional hemisphere. Notably, all network measures in our study tended to be significantly decreased in the ipsilesional postcentral gyrus. This would suggest that the postcentral gyrus, which is greatly involved in somatosensory input, plays a decreased functional role in improving motor function.

The functions of the rostral, caudal, ventral, and dorsal anterior cingulate cortex are reported to be, respectively, execution, assessment, emotion, and recognition [42]. The anterior cingulate cortex connects with motor-related areas including prefrontal cortex and parietal lobe, and is known to play a central role in processing of stimulation or assignment of control to each region in the brain. It is reported to be associated with learning, especially in the initial stage, or problem solving [43]. Our study results indicate that neural network connectivity in the caudal anterior cingulate cortex tended to be decreased in the contralesional hemisphere both in patients who received a combination of LF-rTMS and intensive-OT.

The cingulate gyrus plays a role in connecting regions of the limbic system, and is involved in emotion, information processing, learning, and memory [44]. A narrow area called the isthmus of cingulate gyrus is located posteriorly back along the splenium of corpus callosum. As all network measures tended to be significantly increased in the ipsilesional isthmus of the cingulate gyrus in in patients who received a combination of LF-rTMS and intensive-OT, we speculated that this gyrus may play a large role in motor function recovery.

Rehme et al. [45], in an fMRI study in post-stroke patients with upper limb paralysis, investigated changes in the connectivity between the two cerebral hemispheres in the post-stroke acute phase (≤72 h), sub-acute phase (2 weeks), and chronic phase (3–6 months), and showed that as the upper limb motor function recovered after stroke onset, the affected primary motor cortex had a decreased influence on the contralesional primary motor cortex in the acute phase, the contralesional primary motor cortex had an increased influence on the ipsilesional primary motor cortex in the sub-acute phase, and the ipsilesional primary motor cortex had a negative impact on the contralesional primary motor cortex, and its activity was normalized in the chronic phase. However, in the absence of sufficient recovery of motor functions, the negative impact of the contralesional primary motor cortex on the ipsilesional primary motor cortex was intensified in the chronic phase. Their study [45] found that connectivity was restored in the ipsilesional cerebral hemisphere during the process of recovery from motor impairment after stroke and concluded that the shift from the supporting role of the contralesional primary motor cortex to the inhibitory role of the ipsilesional primary motor cortex formed the basis for applying noninvasive brain stimulation to the contralesional primary motor cortex. Takekawa et al. [46] analyzed the results of single photon emission computed tomography (SPECT) conducted before and after combined LF-rTMS and intensive-OT in patients four or more years after the onset of stroke (57.3 ± 45.9 months). Their results showed that therapy in patients with recovered upper limb function had largely decreased blood flow in the ipsilesional superior frontal and middle frontal areas in comparison with blood flow in the contralesional hemisphere. Although their study [46] showed the correlation between LF-rTMS and intensive-OT-associated improvement of upper limb function and increased blood flow in the contralesional cerebral hemisphere, it did not explain how changes in connectivity, as shown by Rehme et al. [45]. correlate with cerebral blood flow increase or decrease.

Our upper extremity function (FMA and ARAT) results showed that patients with left-sided paralysis had a significant improvement in upper limb function, and network measures in the motor-related area of the affected hemisphere were clearly increased. Especially, our study showed that the functional role of the ipsilesional isthmus of the cingulate gyrus was enhanced in patients who received a combination of LF-rTMS and intensive-OT, which was characteristic in the group. Furthermore, it should also be noted that the connectivity of the structural neural network was increased in several regions of the ipsilesional hemisphere during motor function recovery, and decreased in the contralesional hemisphere. These findings are consistent with those in some prior studies [21,24,39].

Finally, in contrast with the study by Wanni Arachchige et al. [24], our study notably showed the existence of cerebral regions with a significant change in all network measures. In this regard, we could conclude that our study findings are more reliable.

Our study had several limitations. Firstly, our sample size was less than 10 patients in each group and each subgroup, and the number of cases was variable. In fact, the lack of significant change in network measures in patients with right-sided paralysis was probably due to the small number of cases. Secondly, our study had no control group. Therefore, the influence of LF-rTMS could not be completely separated from the therapy of combined LF-rTMS and intensive-OT, and it was difficult to clarify which factor by definition had a greater impact. Despite these critical issues, the results of our study were highly beneficial.

## 5. Conclusions

We analyzed measures of structural neural network connectivity using graph theory to investigate changes in the structural neural networks associated with rehabilitation in patients who received a combination of LF-rTMS and intensive-OT. Patients with left-sided paralysis showed a significant increase in the structural neural network connectivity in the ipsilesional isthmus of the cingulate gyrus, with a significant decrease in connectivity to the ipsilesional postcentral gyrus. These study findings indicate that the use of LF-rTMS and intensive-OT combined in maintenance rehabilitation may enhance functional roles in the motor-related areas of the ipsilesional cerebral hemisphere during motor function recovery after stroke. However, some factors (small sample size, no control group) greatly restrict the final interpretation of our study results.

## Figures and Tables

**Table 1:** Clinical and demographic characteristics of the patients.

	Left Hemiplegia *n* = 9	Right Hemiplegia *n* = 5
Age at intervention, Mean ± SD (years)	59.3 ± 12.0	61.2 ± 14.0
Gender, *n* (%)		
Female	4 (44)	2 (40)
Male	5 (56)	3 (60)
Duration after stroke, Mean ± SD (days)	1064.6 ± 956.4	1434.4 ± 1865.0
Subtype of stroke, *n* (%)		
Cerebral hemorrhage	5 (56)	5 (100)
Cerebral infarction	4 (44)	0 (0)
Brunnstrom stage, *n* (%)		
Upper limb		
III	0 (0)	3 (60)
IV	3 (33)	1 (20)
V	6 (67)	1 (20)
Hand and fingers		
III	3 (33)	4 (80)
IV	3 (33)	0 (0)
V	3 (33)	1 (20)

**Table 2:** The FMA-UE and ARAT scores.

	Left Hemiplegia *n* = 9	Right Hemiplegia *n* = 5	All Patients *n* = 14
FMA-UE scores			
Before rehabilitation	44.8 ± 14.1	24.0 ± 17.4	37.4 ± 17.9
After rehabilitation	49.2 ± 11.2	28.2 ± 17.9	41.7 ± 16.8
(*p*-value)	(*p* = 0.0141)	(*p* = 0.0579)	(*p* = 0.0016)
ARAT scores			
Before rehabilitation	26.6 ± 17.1	9.0 ± 11.8	20.3 ± 17.3
After rehabilitation	29.6 ± 18.0	11.0 ± 12.7	22.9 ± 18.3
(*p*-value)	(*p* = 0.0220)	(*p* = 0.3710)	(*p* = 0.0088)

**Table 3:** Network measures in brain regions significantly changed by rehabilitation therapy.

	Left Hemiplegia	Right Hemiplegia
Degree centrality Nodal efficiency	Ih banks of superior temporal sulcus * Ih isthmus of cingulate gyrus * Ih paracentral lobule * Ih postcentral gyrus *	(No significant change)
Betweenness centrality	Ch caudal anterior cingulate cortex * Ih banks of superior temporal sulcus * Ih isthmus of cingulate gyrus * Ih postcentral gyrus *	(No significant change)
Nodal clustering coefficient	Ch caudal anterior cingulate cortex * Ih isthmus of cingulate gyrus * Ih postcentral gyrus **	(No significant change)

Green characters represent regions with increased value after therapy; blue letters correspond to regions with decreased value after therapy. Ih, ipsilesional hemisphere; Ch, contralesional hemisphere. * *p* < 0.05, ** *p* < 0.01.

## Data Availability

The data presented in this study are available on request from the corresponding author. The data are not publicly available due to privacy restrictions.
